# Integrated Bioinformatics and Experimental Analysis Revealed Crosstalk Between IL-6, Autophagy, Ubiquitination, and Key miRNAs in Female Infertility: Insights from Ovarian Endometriosis and Polycystic Ovary Syndrome

**DOI:** 10.3390/cells14211693

**Published:** 2025-10-28

**Authors:** Saber Nahdi, Maria Arafah, Abdel Halim Harrath

**Affiliations:** 1 Department of Zoology, College of Science, King Saud University, Riyadh 11451, Saudi Arabia; nehdisabeur@gmail.com; 2 Department of Pathology, College of Medicine, King Saud University, Riyadh 11451, Saudi Arabia; marafah83@gmail.com

**Keywords:** female infertility, ovary, interleukin-6, LC3, ubiquitination, miRNAs

## Abstract

Female infertility, affecting millions worldwide, involves complex molecular mechanisms such as chronic inflammation, impaired cellular death, and protein regulation. This study explores how the cytokine IL-6, the autophagy marker LC3, ubiquitination process, and three miRNAs, miR-146a-5p, miR-9-5p, and miR-9-3p, contribute to the control of ovarian function and female infertility. Two expression profile datasets (GSE199225 and GSE146856) were screened and downloaded from GEO. DEGs were screened using the GEO2R and ggVennDiagram tools. The three miRNAs were retrieved from datasets using the multiMiR tool, and IL6-targeted genes were retrieved from MSigDB. IL6 and miRNA interaction networks were constructed. Further, the cross-correlation of LC3 and ubiquitination with the DEGs associated miRNAs was demonstrated. Meanwhile, GO/KEGG pathway enrichment analyses and molecular network interaction analysis were performed. Lastly, immunohistochemistry and quantitative PCR (qPCR) were used to confirm the expression of IL6, LC3, and miRNA in ovarian endometrial tissues compared to control tissues. The results showed that IL-6 drives inflammation in conditions of PCOS and ovarian endometriosis, which then disrupts ovulation and embryo implantation. miR-146a-5p reduced inflammation by targeting the gene TRAF6, while miR-9-5p regulated protein degradation via SQSTM1. In agreement with the bioinformatic approach, experimental analysis revealed reduced IL6 protein expression in ovarian endometriosis tissues while the mRNA IL6 level was increased, suggesting the presence of post-transcriptional regulatory mechanisms that act to limit excessive inflammation, probably through miRNAs. Indeed, the levels of miR-146a-5, which plays a role in immune modulation and inflammatory signaling, were significantly upregulated. Interestingly, an alteration in autophagic markers revealed by elevated LC3 was also observed. Aligned with these experimental data, bioinformatic analysis showed that autophagy genes LC3 and ATG5 and ubiquitination processes were tightly linked to ovarian health, with disruptions accelerating follicle loss and oxidative damage. In conclusion, the results showed that IL-6, miRNAs, and autophagy processes work together to control inflammation and cellular repair in ovarian disorders. This study opens new avenues for targeted treatments to improve fertility outcomes by connecting molecular networks to clinical insights.

## 1. Introduction

Female infertility affects approximately 10–15% of couples globally, with polycystic ovary syndrome (PCOS), endometriosis, and idiopathic ovarian dysfunction representing the leading causes [1]. These conditions are driven by molecular dysregulation, including chronic inflammation, oxidative stress, and defective autophagy, which impairs folliculogenesis, oocyte quality, and endometrial receptivity [2]. Among inflammatory mediators, interleukin-6 (IL-6) is a well-established cornerstone of the chronic low-grade inflammation characteristic of PCOS. Elevated serum and follicular fluid levels of IL-6 are consistently reported in women with PCOS and are strongly correlated with insulin resistance, hyperandrogenism, and dysfunctional ovulation [3,4,5,6]. In endometriosis, IL-6 drives a pro-inflammatory microenvironment that promotes lesion survival and impairs endometrial receptivity [5].

MicroRNAs (miRNAs), small non-coding RNAs, are emerging as master regulators of reproductive health [7,8,9]. MicroRNAs (miRNAs) have emerged as critical post-translational regulators of the pathways disrupted in PCOS and endometriosis. Specifically, miR-146a-5p is recognized for its role in dampening the innate immune response. In PCOS, miR-146a-5p is dysregulated and has been shown to target key genes like TRAF6 and IRAK1 in granulosa cells, directly linking it to the inflammatory signaling prevalent in this syndrome [10,11,12]. Similarly, the miR-9 family (miR-9-3p/5p) is involved in granulosa cell apoptosis and follicular atresia. Studies indicate that miR-9 influences aromatase expression and estrogen production, processes fundamental to follicular development that are impaired in PCOS [13,14,15]. Despite their significance, the interplay between miRNAs, IL-6, and downstream pathways of autophagy and ubiquitination remains underexplored.

Autophagy, a cellular clearance mechanism marked by the conversion of LC3-I to LC3-II, is crucial for maintaining oocyte quality and granulosa cell function. Growing evidence indicates that autophagy is impaired in PCOS ovaries, contributing to oxidative stress and the accumulation of damaged organelles, which in turn exacerbates metabolic and reproductive dysfunction [16,17,18]. The ubiquitination system works in concert with autophagy to regulate protein turnover, and its dysregulation is increasingly implicated in ovarian aging and PCOS pathogenesis [19,20,21]. Dysregulation of these processes is linked to oxidative damage, mitochondrial dysfunction, and apoptosis of the hallmarks of ovarian aging and implantation failure [22].

Current research on female infertility primarily examines isolated pathways, neglecting the integrative roles of IL-6, miRNAs, autophagy, and ubiquitination. A systems-level approach is needed to unravel their crosstalk and identify therapeutic targets. This study hypothesizes that IL-6-driven inflammation, miRNA dysregulation, and impaired autophagy–ubiquitination networks synergistically contribute to female infertility. By leveraging bioinformatics analyses of gene expression datasets GSE199225 [23] and GSE146856 [24], we aim to map these interactions. These datasets were selected for their focus on PCOS, their utilization of high-resolution RNA-seq technology, and their well-matched control groups, providing a robust foundation for our analysis. The results obtained by using bioinformatic tools were confirmed by experimental analysis using immunohistochemistry and quantitative PCR (qPCR) techniques.

While our primary bioinformatic analysis utilized PCOS datasets to map this network, we subsequently sought to explore its potential relevance in the context of ovarian endometriosis, another prevalent condition characterized by inflammation and ovarian dysfunction. This approach aims to identify convergent molecular pathways across different etiologies of female infertility.

## 2. Materials and Methods

### 2.1. Data Processing

The gene expression profiles related to female infertility were retrieved and downloaded from the Gene Expression Omnibus (GEO) public repository of the National Center for Biotechnology Information (NCBI). The retrieved Boolean queries included keywords “Interleukin-6” OR “IL-6”, “LC3” OR “MAP1LC3”, “Ubiquitination” OR “Proteasome pathway”, “miR-146a-5p” OR “miR-9-1”, and the conditions related to female infertility were “Endometriosis” OR “PCOS” OR “Ovarian Dysfunction”. To ensure high-quality data, datasets were prioritized, retrieved, and downloaded using Expression Profiling by using Array or RNA-seq technologies, which provide comprehensive annotations.

GSE199225 [23] is an RNA-seq expression profile based on GPL24676 Illumina NovaSeq 6000 (Homo sapiens) and contains samples of six healthy lean control women, all aged between 18 and 40 years and with PCOS. We specifically selected the GSE199225 dataset, whose data are derived from skeletal muscle, due to the well-established systemic nature of PCOS, characterized by widespread metabolic and inflammatory dysregulation. The transcriptomic profile of muscle in individuals with PCOS reflects this systemic condition and may help identify consistently altered pathways that extend beyond the ovaries. This approach aims to capture a broader range of systemically relevant network dysregulations. GSE146856 [24] is an expression profile-based dataset based on GPL20795 HiSeq X Ten (Homo sapiens) provided by the International Peace Maternity and Child Health Hospital, Shanghai, China, and it contains samples of control tissue and PCOS tissue. The samples of the two expression profile datasets were classified and analyzed based on PCOS.

**Figure 1:**
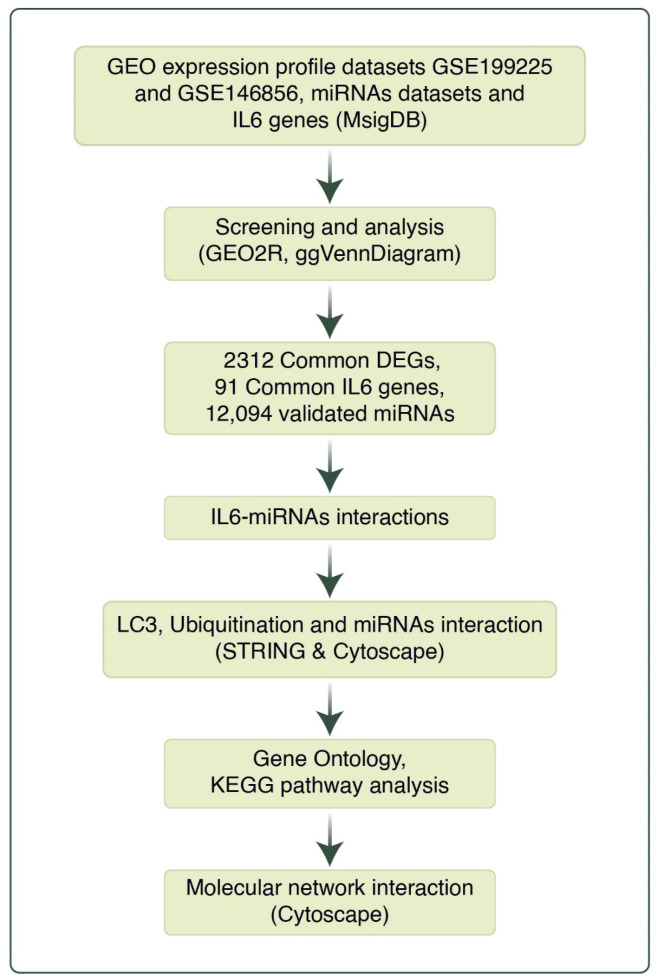
Flowchart of integrated analysis in study.

IL6 signaling-related genes were extracted from the Molecular Signature Database (MSigDB) according to the Human collection Biocarta, Apoptosis, and IL6 pathway gene sets category [25]. microRNAs (miRs) are fundamental regulators of protein-coding genes [26]. miR-146a-5p is a small, non-coding RNA molecule involved in the post-transcriptional regulation of gene expression [27]. miR-9 is a microRNA (miRNA) that plays a crucial role in brain development and function. miR-9-5p and miR-9-3p are two single-stranded microRNAs (miR) derived from the same RNA duplex [26]. The three miRs’ targeted protein-coding genes were derived from miRRecords, miRbase, and TarBase databases by using multiMiR R library [28]. The workflow of this study is illustrated in Figure 1.

### 2.2. Analysis of Microarray Datasets

Based on PCOS’s role in female infertility, in the subsequent analysis, samples of each dataset were defined into two groups: “Control” and “PCOS”. Raw expression data and metadata were extracted by using GEO2R [29,30]. Normalization and log2 conversion were carried out for each dataset to filter out the DEGs, which are displayed as volcano plots. The filtering conditions were as follows: |log2-fold change| ≥ 1 and adjusted *p*-value (adj. P) < 0.05. The ggVennDiagram R package version 1.5.2 was used to compare and analyze the intersection of genes [31]. Based on the intersection results, the final DEGs were obtained and integrated with the results for further analysis.

### 2.3. Identification of miRNA–Target Interactions

To identify high-confidence genes targeted by the three miRNAs miR-146a-5p, miR-9-5p, and miR-9-3p, we utilized the multiMiR R package 0.98.0.2 [28] which provides a comprehensive compilation of miRNA–target interactions from multiple validated and predicted datasets. To ensure high reliability, the analysis was restricted to experimentally validated targets from curated sources within multiMiR, including miRTarBase, TarBase, and miRRecords. The multimir function was used with the parameters table = ‘validated’ and predicted.site = ‘conserved’ to retrieve a list of target genes for each miRNA supported by experimental evidence. The resulting gene symbols for each miRNA were then used for subsequent analyses. ENTREZID IDs were mapped for these target gene lists using the org.Hs.eg.db database to facilitate KEGG pathway enrichment analysis using the clusterProfiler package [32] with the following parameters: organism (hsa), adjusted *p*-value cutoff (pvalueCutoff = 0.05), and Benjamini–Hochberg correction (pAdjustMethod = “BH”). Enriched pathways were filtered for relevance to IL6, autophagy, and ubiquitination, and the results were visualized using dot plots with significance thresholds (qvalueCutoff = 0.05).

### 2.4. Analysis of IL6 and miRNA Interactions

A total of 648 genes were retrieved. A total of 566 unique IL6 genes were found after the deletion of duplicated genes. DEGs with female infertility were first cross-referenced with IL6-related genes obtained from the MSigDB database to identify overlapping genes potentially regulated by IL6 signaling. The overlapped IL6 genes interacted with the three miRNAs’ targeted genes. To examine the interactions more broadly, a relationship network model was constructed between the DEGs associated with IL6 genes and the targeted miRNA genes using Cytoscape version 3.10.3 [33]. Enrichment analysis of the key genes from the network was performed for the interacted genes using Enrichr [34]. Kyoto Encyclopedia of Genes and Genomes (KEGG) pathway [35] and GO enrichment analyses were used to identify the significant pathways.

### 2.5. Analysis of miRNA Interactions with LC3 and Ubiquitination

Autophagy-related genes were curated based on their roles in LC3-mediated autophagy and ubiquitination, according to a literature study. Overlapping targets of miR-146a-5p, miR-9-3p, and miR-9-5p were identified using the multiMiR package [28]. Gene symbols were mapped to Entrez IDs via the org.Hs.eg.db database, and GO Biological Process (BP) enrichment was performed with clusterProfiler using Benjamini–Hochberg correction, where pAdjustMethod = “BH” and ont = “BP” [32]. Significant terms were visualized via dot plots with a pvalueCutoff = 0.05.

### 2.6. Construction of LC3, Ubiquitination, and miRNA Network

To visualize miRNA–gene interactions and their role in autophagy and ubiquitination, a regulatory network was constructed by using Cytoscape [33]. Signaling pathways of upstream regulators of MTOR, TRAF6, and NF-κB were added to the network to show the indirect effect of the genes. The signaling-related genes were downloaded from STRING-DB [36]. While STRING-DB identified broader interactions within the NF-κB pathway, it focused on TRAF6 → NFKB1 → ULK1 due to their direct roles in autophagy regulation and their relevance to female infertility [37,38]. In the style panel, miRNA nodes with red triangles, orange diamond shapes, and dashed line edges were added to the signaling regulators, and all of the gene targets were added in a blue circle with solid line edges.

### 2.7. Pathway Analysis of LC3–Ubiquitination and miRNAs

The protein-coding genes from the autophagy, ubiquitination, and miRNA network were uploaded to Enrichr [34]. The organism selected was Human. The KEGG pathways, Reactome pathways, and GO Biological Processes databases were selected to analyze the pathways. The database generated pathways along with *p*-values and adjusted *p*-values. The top pathways were sorted based on the *p*-values and adjusted *p*-values.

### 2.8. qRT-PCR

A group of women with ovarian endometriosis was confirmed histologically based on formalin-fixed paraffin-embedded (FFPE) tissues. FFPE tissue samples were acquired by using a rotary microtome, enabling the visualization of a single cell layer. Genomic DNA and RNA were extracted from patients with endometriosis and controls using a commercial kit (AllPrep DNA/RNA FFPE kit, Qiagen, Hilden, Germany). All clinical data were used anonymously to protect patient privacy. The quantity and quality of DNA and RNA were measured using a Nanodrop ND-2000C spectrophotometer (Thermo Scientific, Wilmington, DE, USA). The extracted nucleotides were kept at −20 °C until further analysis. Following the manufacturer’s protocol, total RNA (2000 ng/μL) was transcribed into cDNA using a high-capacity cDNA Reverse Transcription Kit (Applied Biosystems 4368814, Carlsbad, CA, USA). Optimal amplification was achieved using qRT-PCR. Primers were designed manually to generate amplicons of ≤120 bp. Each primer was at least 18 nucleotides in length with an appropriate GC content to minimize the formation of secondary structures. To avoid primer dimer formation, both forward and reverse primers were designed with minimal 3′ end complementarity and equal melting temperatures (Tm). Primer specificity was confirmed through BLAST analysis. The oligonucleotide primers (Table 1) were synthesized by Macrogen, Inc. (Seoul, Republic of Korea) and diluted in DNase/RNase-free water to a final working concentration of 10 pmol. qRT-PCR reactions were prepared with Universal SYBR Green Supermix (1725120, Bio-Rad, Hercules, CA, USA). Each well of the 96-well plate contained 10 μL of reaction mix, composed of 4 μL SYBR Green Supermix, 1 μL ROX, 2 μL cDNA, 1 μL of each primer, and 2 μL nuclease-free water. Amplification was performed on a QuantStudio 7 Flex Real-Time PCR System (Applied Biosystems, Hercules, CA, USA). All reactions were run in triplicate. GAPDH served as the reference gene for normalization. Data analysis was performed using SPSS software (version 20; SPSS Inc., Chicago, IL, USA).

### 2.9. Immunohistochemical Staining (IHC)

Sections obtained from FFPE tissue samples and mounted on coated slides were deparaffinized and immersed in xylene twice for 15 min each, rehydrated using a graded alcohol series, and washed in distilled water. The slides were heated in a citric acid buffer for 5 min in a microwave for antigen retrieval. The sections were then rinsed in distilled water to suppress endogenous peroxidase activity and subsequently treated for 5 min with a 0.3% hydrogen peroxide solution. Afterward, 0.01 M phosphate-buffered saline PBS (pH 7.2) was used to rinse the sections. The sections were then incubated with a blocking solution for an hour to minimize non-specific background staining. Next, the primary antibodies (anti-IL6 (ABCAM, ab9324), anti-LC3 (ABCAM, ab229327), and anti-actin (ABCAM, ab1801)) were applied to the samples at 4 °C in a wet chamber overnight. After washing with PBS, the slides were incubated with the corresponding secondary antibody (ABCAM, ab6728) for one hour at room temperature. Hematoxylin was used as a counterstain to provide the stained antigen with a contrasting background. The slides were dehydrated, and xylene was used to clear the sections after passing through a graded alcohol series.

### 2.10. Statistical Analysis

GraphPad Prism software (GraphPad Software version 10.2, San Diego, CA, USA) was used to compare gene and protein expression levels between the control and patient groups. *p*-values less than 0.05 were considered statistically significant.

### 2.11. Ethical Approval

This study was approved by the Ethical Committee and Institutional Review Board of the College of Medicine Research Center at King Saud University Medical City (reference number E-22-6750).

## 3. Results

### 3.1. Identification of DEGs

**Figure 2:**
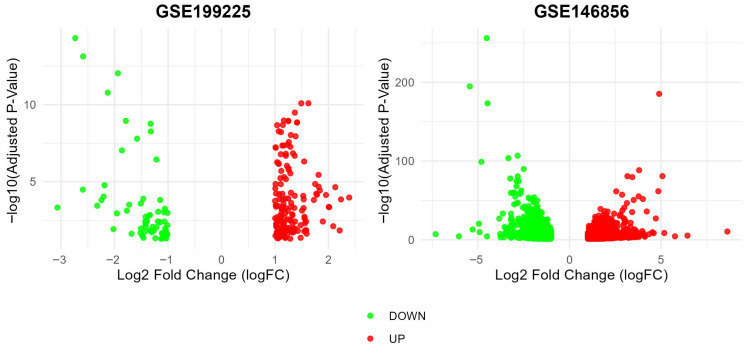
Volcano plot of differentially expressed genes in GSE199225 and GSE146856: Identification of upregulated and downregulated genes.

**Figure 3:**
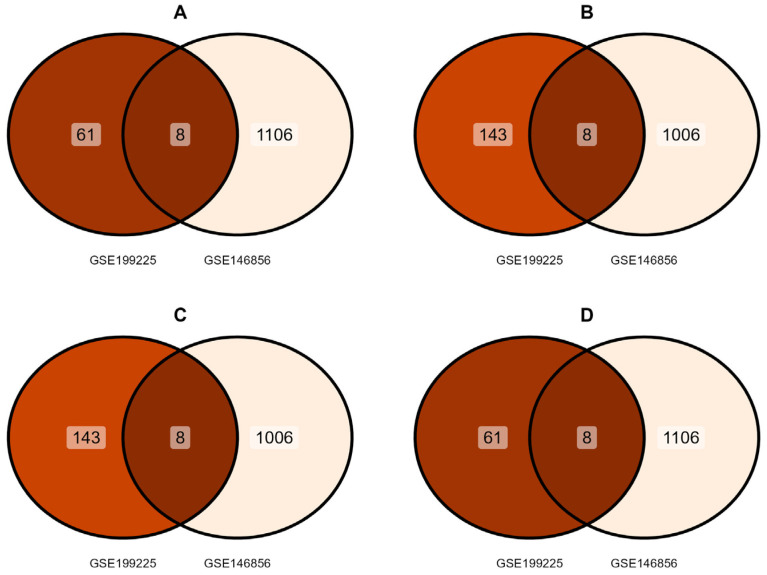
DEG screening via intersection analysis. (**A**) Upregulated genes and (**B**) downregulated genes in control group. (**C**) Upregulated genes and (**D**) downregulated genes in PCOS group.

The correlation of all DEGs from the two expression profile array datasets is shown in the volcano plots (Figure 2). After screening and comprehensive analysis with the ggVennDiagram, eight upregulated and downregulated genes were identified in the control group (Figure 3A,B). Similarly, eight upregulated and downregulated genes were identified in the PCOS group (Figure 3C,D). PSG4, CFI, CHI3L1, BDKRB2, GUCY1A2, SFRP4, IL33, and PADI2 were upregulated in the control group but downregulated in the PCOS group in both datasets, and PITX1, ISG15, NEURL1, OAS2, PRSS3, PI16, L3MBTL4, and MX2 were downregulated in the control group but upregulated in the PCOS group in both datasets. Finally, a total of 2312 DEGs were screened (|log2-fold change| ≥ 1 and adjusted *p*-value (adj. P) < 0.05) after the deletion of duplicated genes, including 1148 upregulated and 1164 downregulated expression genes.

### 3.2. miR Gene Target Pathway Analysis

**Figure 4:**
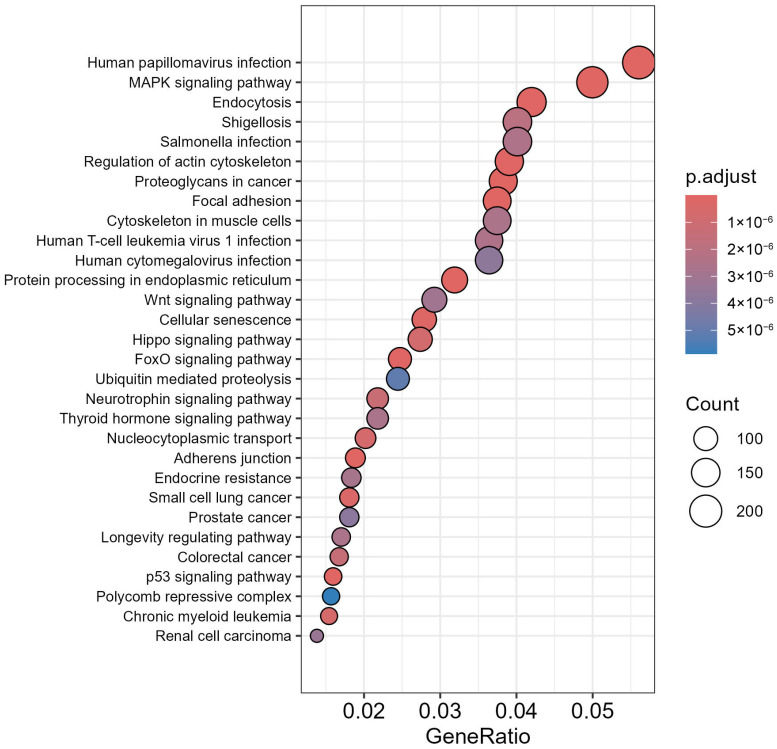
KEGG pathway enrichment analysis of the predicted target genes for miR-146a-5p, miR-9-3p, and miR-9-5p. The analysis reveals significant associations with pathways related to inflammation, autophagy, and ubiquitination.

KEGG enrichment analysis of the combined target genes of miR-146a-5p, miR-9-3p, and miR-9-5p revealed significant associations with pathways central to inflammation, autophagy, and ubiquitination (p.adjust ≤ 5 × 10^−6^). The MAPK signaling pathway (p.adjust = 1 × 10^−6^), linked to IL6-driven inflammation and follicular dysfunction, was the most enriched, followed by ubiquitin-mediated proteolysis (p.adjust = 2 × 10^−6^), implicating miRNA regulation of protein degradation in ovarian aging (Figure 4). Pathways such as FoxO signaling (p.adjust = 4 × 10^−6^) and protein processing in the endoplasmic reticulum (p.adjust = 3 × 10^−6^) highlighted autophagy’s (LC3-associated) roles in endometrial stress responses. Notably, cellular senescence (p.adjust = 5 × 10^−6^) and p53 signaling (p.adjust = 5 × 10^−6^) tied miR-9-3p/5p to oxidative stress and apoptosis in ovarian follicles. IL6-associated pathways (e.g., focal adhesion, cytoskeleton regulation) were enriched (GeneRatio = 0.03–0.05), with miR-146a-5p targeting genes in endocytosis and proteoglycans in cancer, suggesting roles in immune dysregulation and implantation failure.

### 3.3. Identification of IL-6–miRNA Interactions

**Figure 5:**
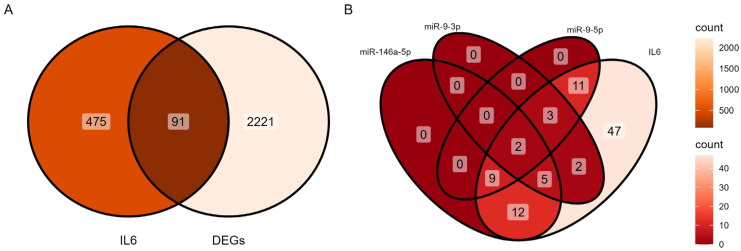
Interaction between DEGs, miRNAs, and IL6 signaling. (**A**) IL6 targeted genes in DEGs. (**B**) DEGs overlapped IL6 genes in miRNAs.

**Figure 6:**
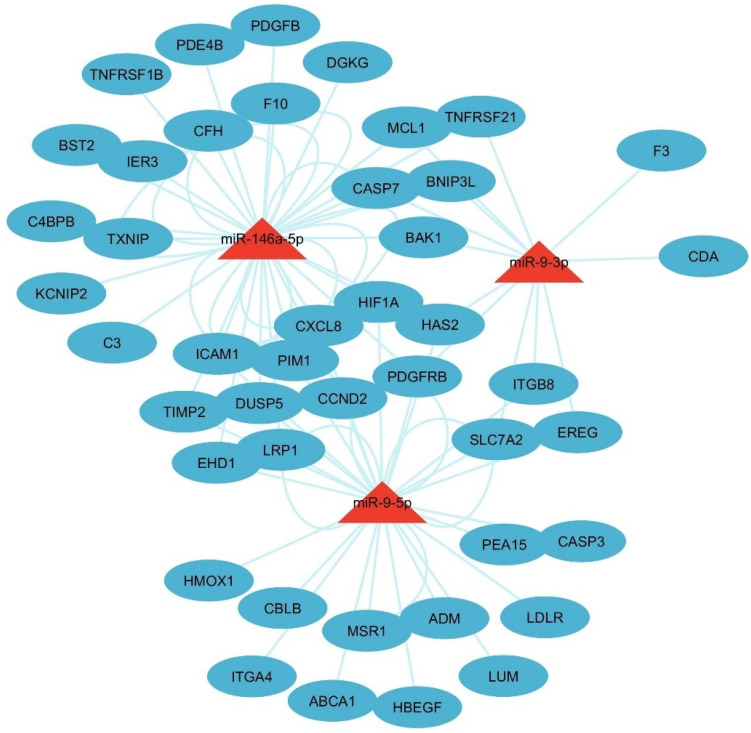
A regulatory network of miRNAs and their target genes within the IL-6 signaling pathway that are dysregulated in female infertility. The model shows how miR-146a-5p, miR-9-3p, and miR-9-5p potentially regulate the shared IL-6/DEG targets.

A total of 566 IL6 genes and 2312 DEGs were intersected and 91 common genes were intersected (Figure 5A). The Venn diagram demonstrates that the 91 IL6 genes matched with the 3 miRNAs, demonstrating the relationship between IL6 and miRNAs (Figure 5). No miRNAs were exclusive to IL6 alone (Figure 5B). This indicates that IL6 interacts with these miRNAs in combination with other pathways. The largest overlap occurring between mir-146a-5p and IL6 involves 28 genes (CFH, CASP7, CCND2, ICAM1, CXCL8, BAK1, TNFRSF21, HIF1A, PIM1, BNIP3L, C3, C4BPB, DUSP5, F10, HAS2, LRP1, PDGFB, PDGFRB, TIMP2, IER3, EHD1, PDE4B, TNFRSF1B, DGKG, BST2, KCNIP2, TXNIP, MCL1), which suggests strong co-regulation between this miRNA and IL6. A core set of two genes, HAS2 and PDGFRB, was shared among IL6, mir-146a-5p, mir-9-3p, and mir-9-5p, highlighting their central role in IL6-associated processes like inflammation [46]. Smaller overlaps included 11 genes (CCND2, ICAM1, CXCL8, HIF1A, PIM1, DUSP5, HAS2, LRP1, PDGFRB, TIMP2, and EHD1) shared by IL6, mir-146a-5p, and mir-9-5p. Five genes, PDGFRB, SLC7A2, EREG, HAS2, and ITGB8, were shared by IL6, mir-9-3p, and mir-9-5p. A core set of seven genes, CASP7, BAK1, TNFRSF21, BNIP3L, HAS2, PDGFRB, and MCL1, were shared by IL6 mir-146a-5p, and mir-9-3p. These results emphasize that IL6 collaborates with mir-146a-5p, mir-9-3p, and mir-9-5p through shared regulatory networks, rather than acting independently, to influence cellular responses [47,48]. To visualize the functional relationships, a network model was constructed focusing on the 91 genes that were common to both the IL-6 signaling pathway and the infertility-associated DEGs (Figure 6). IL6-interacting genes include ICAM1, which is related to inflammation [49], TNFRSF21 related to apoptosis [50], and CXCL8 related to immune signaling [51], which are co-regulated by the miRNAs. mir-146a-5p and mir-9-5p strongly overlap with IL6-associated genes like ICAM1 and TNFRSF21, suggesting their role in modulating IL6-driven pathways. mir-9-3p partially overlaps with CXCL8 and DUSP5, linking it to chemokine signaling and stress responses [51,52]. The network reveals shared targets between IL6 and these miRNAs, indicating coordinated regulation of inflammation and immune processes. mir-146a-5p and mir-9-5p directly targeted IL6-regulated genes, while mir-9-3p co-regulated CXCL6 and DUSP5.

Several key genes in the network model (Figure 6) are associated with female infertility. HAS2 has a critical role for cumulus cell expansion during ovulation [53]. HBEGF is essential for embryo implantation and endometrial receptivity [54]. ICAM1 is linked to endometriosis-associated inflammation and infertility [49,55]. HIF1A influences placental development and abortion [56]. CASP7 regulates apoptosis in ovarian follicles, affecting ovarian reserve [57]. Also, CXCL8 modulates inflammatory processes during ovulation [58].

### 3.4. Enrichment Analysis of Key IL6 Genes

**Figure 7:**
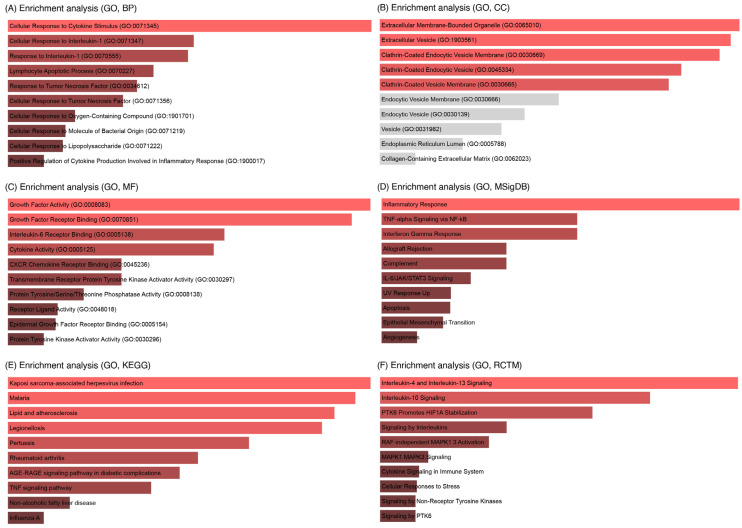
Key IL6 genes associated with apoptosis, female infertility, and miR targeted genes in enrichment analysis. (**A**) Biological process. (**B**) Cellular components. (**C**) Molecular functions. (**D**) MSigDB Hallmark pathways. (**E**) KEGG pathways. (**F**) Reactome pathways. Pathways were considered significant at *p* < 0.05 based on Enrichr’s statistical output.

To identify pathways most critical to IL-6–miRNA crosstalk, we focused our enrichment analysis on a subset of nine key IL-6-associated genes (ICAM1, TNFRSF21, CXCL8, DUSP5, HAS2, HBEGF, HIF1A, CASP7, IL6). These genes were selected based on their central position in the interaction network with the three miRNAs (Figure 6) and their established roles in reproductive processes. *p* < 0.05 was considered the cutoff criterion for significant enrichment. The genes revealed significant associations with inflammatory, immune, and developmental pathways critical to female infertility (Figure 7). Key enriched terms included “Cellular Response to Cytokine Stimulus” (GO:0071345) (Figure 7A), “TNF Signaling Pathway” (KEGG, Figure 7E), and “IL6/JAK-STAT3 Signaling” (MSigDB, Figure 7D), highlighting their roles in inflammation, endometrial dysfunction, and folliculogenesis. Genes such as ICAM1, HAS2, and HBEGF were linked to extracellular matrix remodeling and embryo implantation, while HIF1A and CASP7 were tied to hypoxia and ovarian follicle apoptosis. The MSigDB pathway analysis confirmed IL6 as a central hub coordinating these processes.

### 3.5. Identification of LC3 and Ubiquitination Interaction with miRNAs

**Figure 8:**
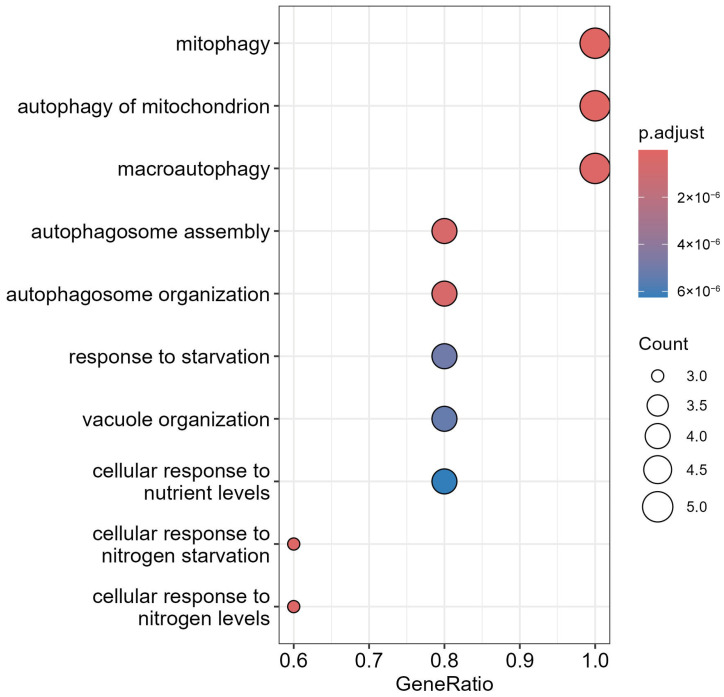
Gene Ontology (GO) enrichment of key autophagy genes.

The curated autophagy-related genes (Table 1) were used to run the GO enrichment analysis, revealing significant associations between miRNA targets and autophagy-related processes, where p.adjust ≤ 6 × 10^−6^ (Figure 8). The top enriched terms were “mitophagy of mitochondrion” with a p.adjust value of 2 × 10^−6^, “macroautophagy” with a p.adjust value of 4 × 10^−6^, and autophagosome assembly with a p.adjust value of 6 × 10^−6^, with a high gene ratio 0.6–1.0. Key genes such as MAP1LC3A and LC3B, which are associated with “autophagosome formation”, SQSTM1 and p62, which are “ubiquitin-binding” genes, and PARK2, which is associated with “mitophagy”, were prominent in these pathways. Additionally, “response to starvation” and “cellular response to nutrient levels” implicated miRNAs in stress-induced autophagy, which is critical for ovarian follicle survival and oocyte quality [59].

### 3.6. Analysis of LC3–Ubiquitination and miRNA Network

**Figure 9:**
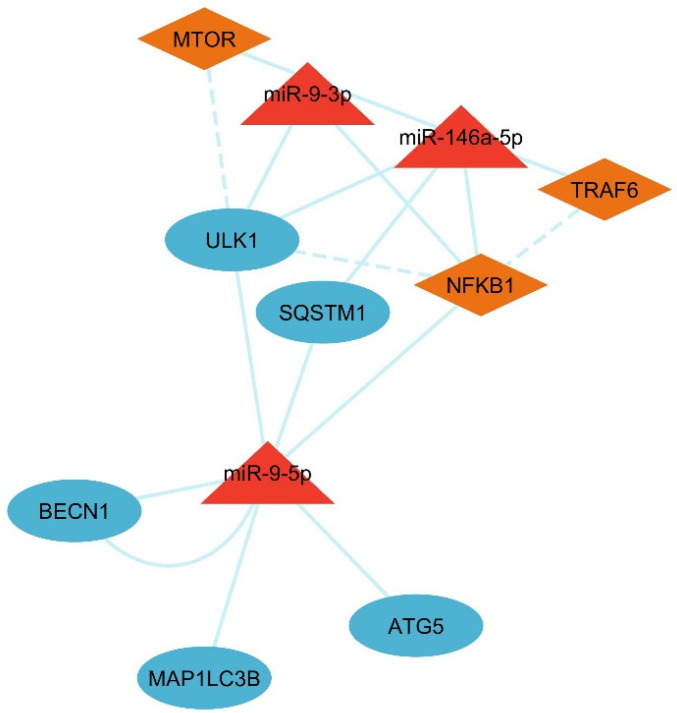
miRNA interactions with autophagy, ubiquitination, and upstream signaling regulators.

Cytoscape network analysis revealed miR-146a-5p, miR-9-3p, and miR-9-5p as central regulators of upstream autophagy and inflammatory signaling (Figure 9). The network highlights cross-regulation between autophagy (ATG5, MAP1LC3B) and inflammatory pathways (TRAF6, NFKB1), with miRNAs acting as molecular bridges. miR-146a-5p suppresses TRAF6 and NFKB1, dampening IL6-driven inflammation while indirectly promoting autophagy via reduced MTOR activation [60,61,62]. miR-9-3p targets MTOR by directly stimulating autophagy initiation through ULK1 and BECN1 [63]. Also, miR-9-5p regulates SQSTM1/p62, linking ubiquitination to autophagosome formation via MAP1LC3B (LC3B) [64].

### 3.7. GO Analysis of LC3–Ubiquitination and miRNA Interactions

**Figure 10:**
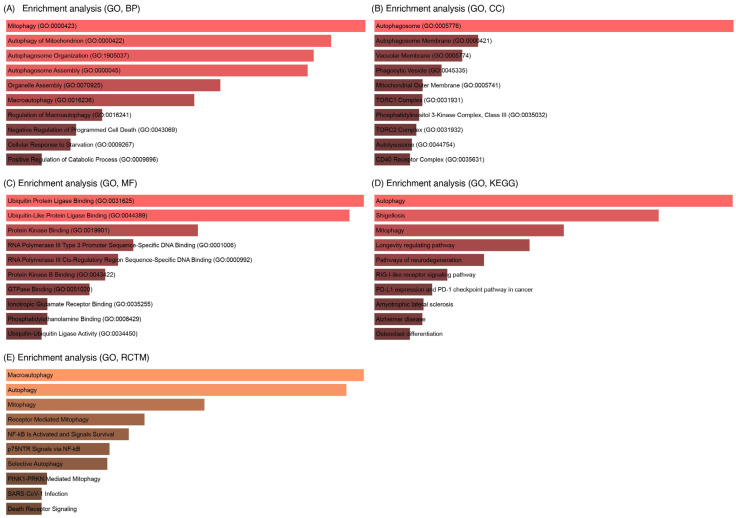
LC3–ubiquitination and miRNA targeted gene enrichment analysis. (**A**) Biological process. (**B**) Cellular components. (**C**) Molecular functions. (**D**) KEGG pathways. (**E**) Reactome pathways. Enriched terms shown met statistical significance thresholds (*p* < 0.05) as calculated by Enrichr.

GO enrichment analysis of upstream signaling networks revealed significant associations between miR-146a-5p, miR-9-3p, miR-9-5p, and pathways critical to autophagy, ubiquitination, and inflammation (Figure 10). Strong enrichment of macroautophagy (GO:0016236) (Figure 10E), autophagosome assembly (GO:0000045) (Figure 10A), and mitophagy (GO:0000423) (Figure 10A) highlights miRNA regulation of mitochondrial quality control and autophagic flux in the biological process. In the cellular component pathway (Figure 10B), autophagosome membrane (GO:0009421) and TORC1 complexes (GO:0031931) were enriched, linking miRNAs to nutrient-sensing pathways (e.g., MTOR) in ovarian follicle survival. Also, ubiquitin ligase binding (GO:0031625) (Figure 10C) and protein kinase binding (GO:0019901) (Figure 10C), implicating miRNAs in ubiquitination and stress signaling, are revealed in the molecular functions. Moreover, in the KEGG and RCTM pathways (Figure 10D,E), autophagy, PINK1-PARKIN-mediated mitophagy, and NF-κB signaling dominated, with PD-1 checkpoint pathways as well as other significant pathways and their overlap genes (Table 2) suggesting immune–infertility crosstalk.

### 3.8. Interaction of LC3, IL6, and Ubiquitination Genes with Key Biological Processes

**Figure 11:**
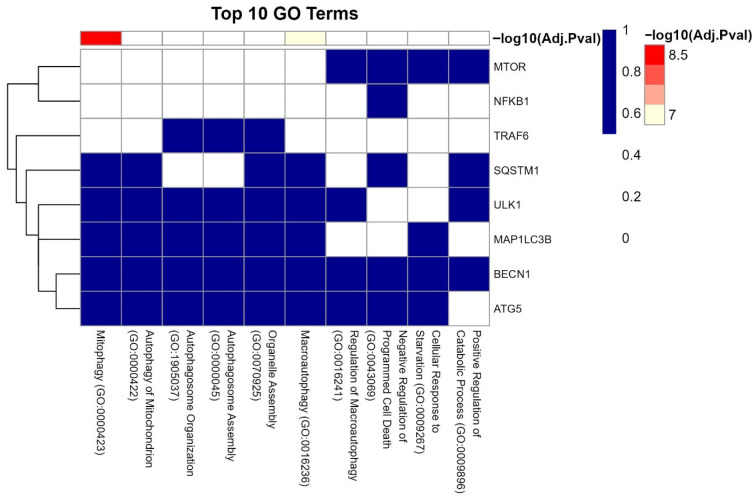
Clustered associations of autophagy, inflammation, and ubiquitination genes with key biological processes.

**Figure 12:**
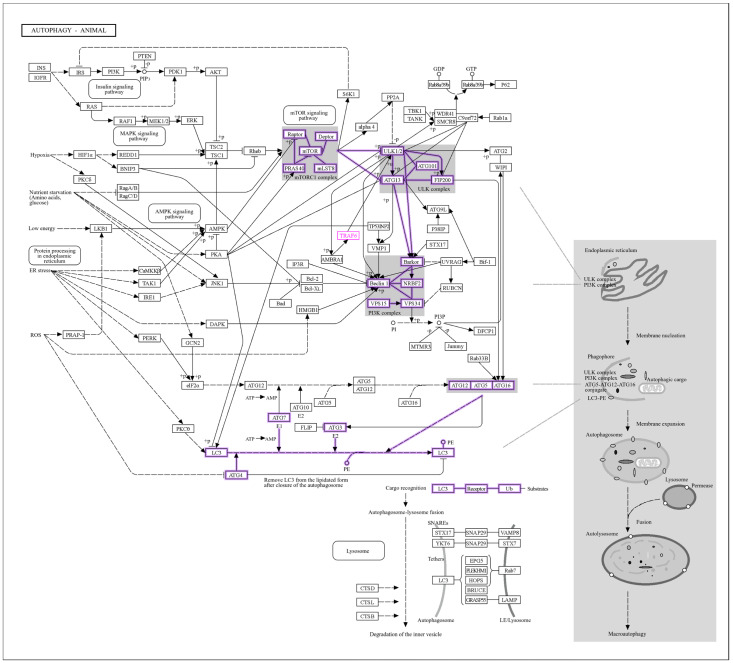
The KEGG autophagy pathway (nt06532). The core molecular machinery of macroautophagy, including the mTOR signaling pathway, which inhibits ULK1 and the LC3 conjugation system. Genes highlighted in purple (ULK1/2, Beclin 1, LC3, ATG5, ATG7) are key targets of miR-9-3p and miR-9-5p identified in this study, demonstrating their role in regulating autophagic flux.

**Figure 13:**
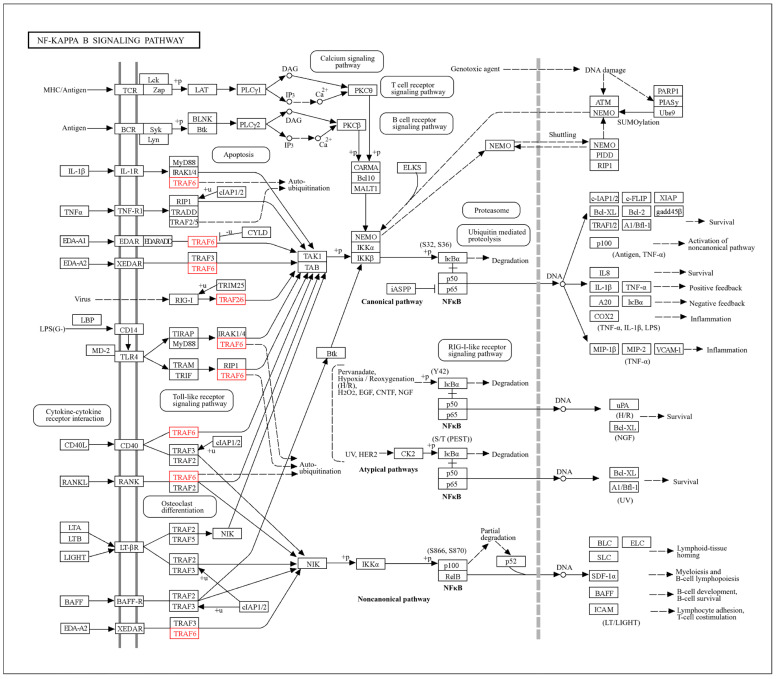
The NF-κB signaling pathway (nt06516). It depicts the canonical and non-canonical NF-κB activation pathways. Genes highlighted in red (TRAF6) represent key nodes targeted by miR-146a-5p, indicating their role in suppressing inflammatory signaling downstream of IL-6 and other cytokines.

The cluster gram (Figure 11) highlights significant associations between IL6–miRNA network genes and autophagy-related biological processes with log10(Adj.Pval) ≥ 0.2–0.8. MTOR exhibited the strongest link to negative regulation of programmed cell death (GO:0043069, log10(Adj.Pval) = 0.8), followed by NFKB1 and TRAF6 with cellular response to starvation (GO:0009267, log10(Adj.Pval) = 0.6) (Figure 12 and Figure 13) [65]. Autophagy genes MAP1LC3B, BECN1, and ATG5 clustered tightly with macroautophagy (GO:0016236) and autophagosome assembly (GO:0000045), underscoring their roles in ovarian follicle survival. SQSTM1/p62 was strongly linked to mitophagy (GO:0000423), emphasizing its role in mitochondrial quality control [66].

### 3.9. Experimental Analysis

**Figure 14:**
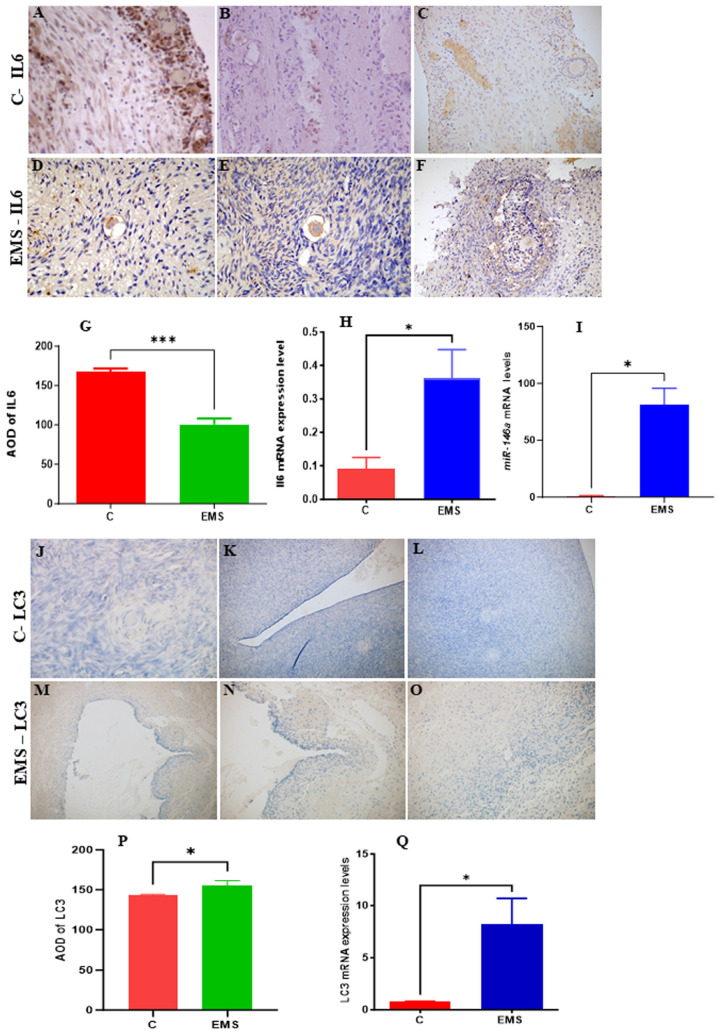
(**A**–**G**) Representative immunohistochemical staining analysis of IL-6 expression in endometrial tissues from control and endometriosis (EMS) groups. Immunohistochemistry showing IL-6 localization predominantly in the cytoplasm and nuclei of glandular and stromal cells. EMS tissues display less strong IL-6 staining (**D**–**F**) compared to control samples (**A**–**C**), indicating reduced protein expression in the patient group. (**H**) qRT-PCR analysis demonstrates a significant increase in IL-6 mRNA levels in EMS tissues relative to controls (*p* < 0.05). (**I**) A significant increase in miR-146a-5 mRNA levels in EMS tissues relative to controls (*p* < 0.05). (**J**–**O**) Representative immunohistochemical staining of LC3 expression in endometrial tissues from control and endometriosis (EMS) groups. Immunohistochemistry showing weak LC3 staining in the control group (**J**–**L**) compared to EMS tissues (**M**–**P**), with faint cytoplasmic localization in glandular and stromal cells. (**Q**) qRT-PCR analysis demonstrates significant upregulation of LC3 mRNA levels in EMS tissues compared to control samples (*p* < 0.05). AOD: average optical density. (*) *p* value < 0.05, (***) *p* value < 0.0001).

We performed analyses of the mRNA and protein expression of IL-6 in ovarian endometriosis. Protein expression analysis using immunohistochemistry (IHC) revealed a paradoxical downregulation of IL-6 protein levels in endometriotic lesions relative to normal (Figure 14A–G), while a significant increase in IL-6 mRNA revealed by quantitative real-time PCR (qRT-PCR) analysis was observed (Figure 14H). The observed discrepancy between IL-6 mRNA and protein expression indicates the possibility of post-transcriptional regulatory mechanisms, including miRNAs. Our findings indicate notable upregulation of miR-146a-5p (Figure 14I), which plays a key role in immune modulation and inflammatory signaling, highlighting the significance of immune dysregulation in disease progression. Interestingly, protein expression analysis using IHC consistently showed a notable increase in LC3 protein levels in ovarian endometriotic lesions compared to control samples (Figure 14J–P), in accordance with the qRT-PCR result which demonstrated a significant increase in LC3 mRNA levels (Figure 14Q). While this upregulation of LC3 indicates an altered autophagic process, we note that without concurrent measurement of autophagic flux (e.g., via p62/SQSTM1 degradation analysis), these data specify dysregulation of the pathway rather than its functional throughput.

## 4. Discussion

Female infertility is a major global health issue, with PCOS and ovarian endometriosis being among the most prevalent endocrine disorders associated with it [1]. Both endometriosis and PCOS impact up to 10% of women, severely affecting their health, fertility, and overall quality of life [67]. PCOS is characterized by hormonal imbalances, chronic inflammation, and metabolic dysregulation, which collectively impair ovulation, endometrial receptivity, and follicular development [4]. While our primary bioinformatic analysis utilized PCOS datasets to map a core molecular network, we subsequently sought to explore its potential relevance in the context of ovarian endometriosis, another prevalent condition characterized by inflammation and ovarian dysfunction. This approach aims to identify convergent molecular pathways across different etiologies of female infertility. In this study, we employed a comprehensive bioinformatics approach and experimental analysis to unravel the molecular crosstalk between interleukin-6 (IL6), autophagy, ubiquitination, and three microRNAs, miR-146a-5p, miR-9-3p, and miR-9-5p, in the context of female infertility. By analyzing gene expression profiles from PCOS and control groups from GSE199225 and GSE146856 datasets, we identified 2312 differentially expressed genes (DEGs), including 1148 upregulated and 1164 downregulated genes. These DEGs were intricately linked to IL6 signaling, autophagy markers (LC3), ubiquitination pathways, and miRNA interactions, revealing a complex regulatory network that underscores the multifactorial nature of infertility [2].

MicroRNAs, small non-coding RNAs that have fine-tuned gene expression, emerged as pivotal regulators in this study. Using the multiMiR database [28], we identified 12,094 miRNA–target interactions, with miR-146a-5p, miR-9-3p, and miR-9-5p targeting 5505, 1359, and 5230 genes, respectively. The KEGG enrichment analysis [35] highlighted their roles in IL6-driven inflammatory cascades, autophagy, and ubiquitin-mediated proteolysis. The MAPK signaling pathway, central to IL6-mediated inflammation, was strongly enriched with miR-146a-5p by suppressing MAPK inhibitors (DUSP5), thereby amplifying inflammatory signals that disrupt folliculogenesis and endometrial function [68]. Concurrently, miR-9-5p regulates ubiquitin ligases (FBXW7) to stabilize LC3-associated autophagic proteins, which is a process critical for clearing damaged cellular components in aging ovarian follicles [19,44,69,70]. These findings position miRNAs as molecular bridges connecting inflammation, autophagy, and protein degradation in reproductive pathophysiology.

Network modeling further elucidated the collaborative roles of IL6 and miRNAs in female infertility. IL6, a pro-inflammatory cytokine that has been reported to be highly expressed in ovarian endometriosis [71], dominated pathways of TNF signaling and NF-κB activation, which are hyperactive in endometriosis and implantation failure [72]. Notably, miR-146a-5p suppressed ICAM1, a gene overexpressed in endometriosis that promotes leukocyte infiltration and tissue damage (Figure 6) [73]. This suggests a feedback loop where IL6-induced inflammation upregulates ICAM1, while miR-146a-5p acts as a compensatory brake, a mechanism likely disrupted in infertility. In accordance with the network modeling findings, experimental analysis using qRT-PCR analysis revealed significant upregulation of IL-6 mRNA levels. However, protein expression analysis using IHC showed paradoxical downregulation of IL-6 protein levels. This inconsistency—elevated mRNA levels but reduced protein expression—may contribute to the persistence of ovarian endometriotic lesions by mitigating excessive inflammation through the suppression of IL-6 protein levels. One potential explanation for this regulation involves miRNAs, which are critical in post-transcriptional gene regulation by inhibiting the translation of target mRNAs [74]. Specifically, numerous studies have demonstrated altered miRNA expression in ectopic and eutopic endometrial tissues [75,76]. Our findings show that miR-146a-5p, which plays a key role in regulating inflammation, immune responses, and autophagy [77], is significantly upregulated in ovarian endometrial tissues compared to controls. Consistent with previous research [78], miR-146a-5p suppresses the production of pro-inflammatory cytokines like IL-6 through post-transcriptional mechanisms. By binding to the 3′ untranslated region of IL-6 mRNA, miR-146a-5p can inhibit translation or promote mRNA degradation, resulting in decreased IL-6 protein expression.

Similarly, miR-9-5p regulated CASP7, an apoptosis executor, linking IL6-driven oxidative stress to accelerated follicular atresia [79]. Extracellular matrix genes HAS2 and HBEGF, essential for ovulation and embryo implantation, were modulated by IL6 and miRNAs. miR-9-3p indirectly influenced HBEGF-mediated embryo attachment by targeting DUSP5, a phosphatase that regulates endometrial signaling [13]. These interactions mirror clinical observations in PCOS, where IL6 overexpression correlates with defective follicle rupture and implantation failure [4].

GO and KEGG enrichment analyses emphasized the centrality of autophagy and ubiquitination in maintaining ovarian and endometrial homeostasis (Figure 8, Figure 10 and Figure 11). Autophagy-related terms “mitophagy” (GO:0000423) and “autophagosome assembly” (GO:0000045) were enriched with genes MAP1LC3B and SQSTM1/p62, which interact with miR-9-3p and miR-9-5p to maintain mitochondrial and protein quality [22]. Dysregulation of these genes, as seen in endometriosis or PCOS, could impair autophagic flux, exacerbating oxidative damage and fibrosis [16]. The NF-κB signaling pathway further connected IL6 to chronic inflammation, while the Autophagy-Animal pathway highlighted LC3-mediated processes critical for ovarian reserve [19]. Cluster analysis (Figure 11) revealed tight associations between IL6-associated genes (MTOR, NFκB1) and autophagy effectors BECN1 and ATG5, demonstrating their convergence on pathways governing follicular atresia and nutrient sensing (Figure 13). Furthermore, MTOR, a master regulator of autophagy (Figure 12), suppressed apoptosis under metabolic stress but inhibited autophagy via ULK1 repression [80]. miR-9-3p’s targeting of MTOR could restore this balance, enhancing oocyte survival in PCOS [13]. Aligned with the bioinformatic analysis, our experimental findings demonstrate significant upregulation of LC3 mRNA and protein levels in ovarian endometriotic tissues, as confirmed through qRT-PCR and IHC. These results align with previous studies that reported increased expression of autophagic markers, such as Beclin-1 and LC3-II, in ovarian endometriomas compared to eutopic and normal endometria, suggesting altered autophagic markers in endometrial tissues [81,82]. 

This dysregulation of autophagy markers likely enables ectopic endometrial cells to survive under adverse conditions, such as oxidative stress and inflammation, by providing a mechanism for adaptation to hypoxic and inflammatory environments. These findings are consistent with earlier research showing that altered autophagy processes support the proliferation and survival of endometrial cells [83]. Therefore, targeting autophagy-related processes could represent a promising therapeutic strategy to disrupt the survival mechanisms of endometriotic cells.

Based on our bioinformatic and experimental findings, we propose a working model for the pathophysiology of female infertility centered on the crosstalk between IL-6, miRNAs, autophagy, and ubiquitination. In this model, chronic inflammation, driven by elevated IL-6, is the initial insult that disrupts the follicular environment and endometrial receptivity. Dysregulated miRNAs act as critical molecular tuners where miR-146a-5p is upregulated as a compensatory brake to suppress IL-6/TRAF6/NF-κB signaling and mitigate inflammation, while the miR-9 family members regulate autophagic (via MTOR & SQSTM1) and apoptotic pathways to manage cellular stress. The interplay between these pathways determines cellular fate. The miR-mediated response influences the autophagy–ubiquitination system (evidenced by LC3 elevation), which is essential for clearing inflammation-induced damage. The paradoxical findings, such as increased IL-6 mRNA but decreased protein, alongside elevated miR-146a-5p and LC3, epitomize this dynamic, failed repair process. The breakdown of this feedback loop results in a detrimental cycle of oxidative stress, impaired oocyte quality, and failed implantation, characterizing conditions like PCOS and endometriosis.

All together, these finding offers critical insights into the collective impact on reproductive health. IL-6, a central driver of chronic inflammation, disrupts ovarian folliculogenesis, endometrial receptivity, and follicular survival, particularly in conditions of endometriosis and PCOS. miRNAs act as molecular tuners, modulating IL-6 signaling to balance inflammation, autophagy, and protein degradation. miR-146a-5p suppresses inflammatory mediators (TRAF6/NFκB), while miR-9-5p regulates ubiquitination (via SQSTM1) to maintain cellular quality. Autophagy, marked by LC3-mediated processes, and ubiquitination emerge as essential mechanisms for mitochondrial health, protein clearance, and oocyte survival processes impaired in infertility. Neutralizing IL-6 (with tocilizumab) may mitigate inflammation-driven endometrial damage and ovarian dysfunction. miR-146a-5p mimics may suppress inflammation and modulate autophagy, while miR-9-3p/5p mimics may restore autophagy–ubiquitination balance to improve oocyte quality. Also, activating LC3-mediated pathways (via rapamycin analogs) may rescue follicular atresia and oxidative stress in aging ovaries.

A limitation of our experimental analysis is the assessment of autophagy solely through LC3 expression. As correctly noted, LC3 accumulation can signify either the induction of autophagy or a blockade in the flux of the pathway. Our bioinformatic data implicate SQSTM1/p62 as a key target within this network, suggesting its involvement. However, future studies employing techniques such as lysosomal inhibition or direct measurement of p62 degradation are required to precisely define the nature of the autophagic defect, whether it is hyperactive, impaired, or blocked, in these infertility disorders.

## 5. Conclusions and Perspectives

This study identifies novel regulatory axes with translational potential. The dual role of miR-146a-5p in suppressing inflammation (via TRAF6/NFκB) and promoting autophagy offers a unique therapeutic avenue for endometriosis [10]. Similarly, miR-9-3p mimics could enhance autophagic clearance in ovarian follicles, while miR-9-5p modulation might stabilize SQSTM1/p62-LC3B interactions to mitigate protein aggregation in aging ovaries [22]. IL6 inhibitors (tocilizumab) combined with miRNA-based therapies could address multifactorial infertility by simultaneously dampening inflammation and modulating autophagy process [84]. While our bioinformatics approach provides robust insights, which have been in part validated by some experimental studies, more experimentation is also required. For instance, luciferase assays could confirm miRNA–gene targeting [85], and in vitro models of ovarian/endometrial cells could clarify tissue-specific effects. Additionally, pathways like “Shigellosis” (Figure 10D) and “SARS-CoV-1 infection”, (Figure 10E) though statistically enriched, lack clear biological relevance to infertility and warrant cautious interpretation [86]. Future studies may prioritize clinical cohorts to validate these networks and explore immune–autophagy crosstalk suggested by terms like “PD-1 checkpoint pathway.”

## Figures and Tables

**Table 1:** Key autophagy/ubiquitination genes and their roles in female infertility.

Gene	Functional Role	Associated Reproductive Process	Reference
MAP1LC3B	Autophagosome membrane formation	Impaired oocyte maturation, embryo quality	[39]
ATG5	Autophagosome elongation (ATG5-ATG12 complex)	Follicular atresia, granulosa cell apoptosis	[40]
BECN1	Autophagy initiation (PI3K complex)	Dysregulated autophagy in endometrial cells	[41,42]
SQSTM1	Selective autophagy of ubiquitinated proteins	Protein aggregate clearance in ovarian cells	[39,43]
ULK1	Master kinase regulating autophagy initiation	Stress response in cumulus cells	[44,45]

**Table 2:** Top 10 significant *p*-values and q-values for protein-coding genes in KEGG/Reactome pathways, 2024.

Term	*p*-Value	q-Value	Overlap Genes
Macroautophagy	3.325892 × 10^−12^	5.372732 × 10^−10^	BECN1, MAP1LC3B, ULK1, SQSTM1, MTOR, ATG5
Autophagy	6.105378 × 10^−12^	5.372732 × 10^−10^	BECN1, MAP1LC3B, ULK1, SQSTM1, MTOR, ATG5
Mitophagy	8.588874 × 10^−10^	5.038806 × 10^−8^	MAP1LC3B, ULK1, SQSTM1, ATG5
Receptor-Mediated Mitophagy	6.919482 × 10^−9^	3.044572 × 10^−7^	MAP1LC3B, ULK1, ATG5
NF-kB Is Activated and Signals Survival	1.198939 × 10^−8^	4.220264 × 10^−7^	TRAF6, SQSTM1, NFKB1
p75NTR Signals via NF-kB	2.346278 × 10^−8^	6.359648 × 10^−7^	TRAF6, SQSTM1, NFKB1
Selective Autophagy	2.529405 × 10^−8^	6.359648 × 10^−7^	MAP1LC3B, ULK1, SQSTM1, ATG5
PINK1-PRKN-Mediated Mitophagy	2.071973 × 10^−7^	4.393258 × 10^−6^	MAP1LC3B, SQSTM1, ATG5
SARS-CoV-1 Infection	2.496169 × 10^−7^	4.393258 × 10^−6^	BECN1, MAP1LC3B, TRAF6, NFKB1
Death Receptor Signaling	2.496169 × 10^−7^	4.393258 × 10^−6^	TRAF6, ULK1, SQSTM1, NFKB1

## Data Availability

The data that support the findings of this study are available from the corresponding author (Abdel Halim Harrath) upon reasonable request.
